# GMSA-Net: A Transmission Line Ice Thickness Identification Network Based on Global Micro Strip Awareness

**DOI:** 10.3390/s24134053

**Published:** 2024-06-21

**Authors:** Yu Zhang, Yinke Dou, Yangyang Jiao, Liangliang Zhao, Dongliang Guo

**Affiliations:** 1 College of Electrical and Power Engineering, Taiyuan University of Technology, Taiyuan 030024, China; zhang.yu.edu@163.com; 2 Department of Automation, Taiyuan Institute of Technology, Taiyuan 030008, China; 3 Shanxi Energy Internet Research Institute, Taiyuan 030032, China; jiaoyyru@163.com (Y.J.); zhaoliangliang_97@163.com (L.Z.); 987hans@163.com (D.G.); 4 Key Laboratory of Cleaner Intelligent Control on Coal & Electricity, Ministry of Education, Taiyuan 030024, China

**Keywords:** transmission lines, image segmentation, ice thickness, deep learning

## Abstract

Ice-covered transmission lines seriously affect the normal operation of the power transmission system. Resonance deicing based on different ice thicknesses is an effective method to solve the issue of ice-covered transmission lines. In order to obtain accurate ice thickness of transmission lines, this paper designs an ice thickness of transmission line recognition model based on Global Micro Strip Awareness Net (GMSA-Net) and proposes a Mixed Strip Convolution Module (MSCM) and a global micro awareness module (GMAM). The MSCM adapts to the shape of ice-covered transmission lines by using strip convolutions with different receptive fields, improving the encoder’s ability to extract ice-covered features; the GMAM perceives through both global and micro parts, mining the connections between semantic information. Finally, the ice thickness of the generated segmented image is calculated using the method of regional pixel statistics. Experiments are conducted on the dataset of ice-covered transmission lines. The mean Intersection over Union (mIoU) of image segmentation reaches 96.4%, the balanced F-Score (F1-Score) is 98.1%, and the identification error of ice thickness is within 3.8%. Experimental results prove that this method can accurately identify the ice thickness of transmission lines, providing a control basis for the application of resonant deicing engineering.

## 1. Introduction

The transmission line is a crucial part of the power transmission system, responsible for transmitting electricity between power plants and electrical equipment, and therefore, its transmission stability is crucial [1,2]. Due to the overhead status of transmission lines, they are easily affected by factors such as climate and terrain. Ice-covered transmission lines in winter are a common hazard. After the transmission lines freeze, the significant increase in the weight of the cables can exceed their load-bearing capacity, causing cable deformation, and potentially leading to the collapse of the towers at both ends of the transmission lines [3,4,5]. Severe ice-covered transmission lines affect the normal operation of the transmission system and bring great inconvenience to daily life. Therefore, real-time monitoring of the status of transmission lines is very important [6,7]. If the ice thickness is accurately identified, it can effectively warn people of the ice-covered problem on transmission lines in winter [8,9]. Therefore, how to accurately identify the ice thickness is an urgent problem that needs to be solved.

At present, there are natural observation methods [10] and physical model methods [11] for identifying the thickness of ice. However, the natural observation method requires a lot of manpower and energy, and the observation results are unreliable. The physical model method calculates the ice thickness by establishing a physical model but cannot achieve real-time detection [12]. The image detection method can meet the requirements of real-time performance and accuracy [13]. By placing an imaging device on the transmission cable and using computer vision technology for image processing, the ice thickness can be identified. The ice thickness recognition methods based on image detection are mainly divided into traditional image methods [14] and deep learning methods [15].

Traditional image recognition methods calculate the geometric, edge, and texture information of images through manually designed feature extraction methods, such as Scale-Invariant Feature Transform (SIFT) [16], Local Binary Patterns (LBPs) [17], Speeded Up Robust Features (SURFs) [18], etc. Nusantika et al. [19] first used image filters to enhance the color information of ice-covered images, and then introduced an enhanced multi-threshold algorithm for ice-covered area segmentation. Wang et al. [20] proposed an ice thickness identification method based on the least squares Hough transform, which combines the Hough transform with the least squares method to identify the edges of the ice-covered transmission lines and then calculate the ice thickness. Hu et al. [21] first used the K-Singular Value Decomposition (K-SVD) algorithm for image denoising, then used pixels to distinguish the ice-covered area, and performed pixel statistics to calculate the ice thickness. Traditional methods perform well in simple scenarios, but they cannot effectively identify ice thickness in complex real-world environments.

In recent years, the continuous development of deep learning technology has gradually replaced traditional image processing methods in the field of computer vision [22]. From Visual Geometry Group (VGG) [23] and Residual Network (ResNet) [24] to Vision Transformer [25], neural networks have demonstrated their powerful feature extraction capabilities. Guo et al. [26] proposed a transmission line ice-covered detection method using local convolutional features and designed a local multi-layer convolutional neural network, which can extract richer features compared to ordinary convolutional neural networks. Wang et al. [27] proposed a lightweight ice thickness detection method that uses light-weight convolutional neural networks for feature extraction, and designed a multi-scale object detection network to extract high-dimensional features. However, its recognition performance for complex scenes is poor. Hu et al. [28] used the U-Net network [29] for ice-covered area segmentation, incorporating an attention mechanism and auxiliary loss, and utilized transfer learning for training. Lin et al. [30] proposed an ice thickness of transmission lines detection network based on strong generalized convolutional neural networks. The number of filters for different convolutional layers is determined via the Grow-And-Prune (GAP) mechanism. Following this, the bidirectional greedy algorithm is employed to identify the optimal model layer. Eventually, the ice thickness is calculated. Pi et al. [31] proposed a deep learning-based ice thickness identification method for transmission lines. Image enhancement technology was used to enhance and expand the dataset of ice-covered transmission line data, and then the Mask RCNN network was used to identify the ice thickness of transmission lines. However, because the method used was an object detection algorithm, only the interval of ice thickness could be identified. It could not be accurately identified. Ma et al. [32] proposed a multi-scale feature fusion-based ice state perception method for transmission lines, which fused a residual neural network and feature pyramid network for feature extraction, and then used a full convolutional neural network for ice thickness identification. This method also divides the ice thickness of transmission lines into different levels for identification.

At present, the technology of using deep learning to detect the ice thickness of transmission lines is mainly divided into three categories: image segmentation, object detection and edge detection. By dividing the ice-covered area and the background area of the transmission line, the image segmentation algorithm can determine the ice-covered area and calculate the ice thickness. The object detection method divides the thickness of the ice-covered transmission line into different intervals and classifies the thickness of the ice-covered transmission line. This method can only obtain the fuzzy range of the ice thickness and cannot calculate the accurate ice thickness. In addition, there are also methods to calculate the ice thickness by using edge detection technology, which can obtain the ice thickness by calculating the distance between the upper and lower edges of the ice-covered transmission line. However, at present, the traditional edge detection algorithms are mainly used, and few researchers use deep learning technology to detect the ice edge. Because the upper and lower edges of the ice-covered area are not neat, the ice thickness calculated by this method is not accurate enough. Therefore, we use image segmentation to identify the ice thickness of transmission lines. Upon analyzing the above-mentioned method of using deep learning to detect the ice thickness of transmission lines, we find that they do not consider the shape characteristics of the ice-covered transmission lines. Additionally, due to the inherent locality of convolution, these methods lack the ability to calculate the global relationship of the image.

We propose a method for identifying the ice thickness of transmission lines based on GMSA-Net. This method obtains ice-covered images of transmission lines through the camera of the deicing robot, then uses image segmentation technology to segment the ice-covered area, and finally calculates the ice thickness. We use global micro strip awareness to obtain the part covered by ice and snow, and in the image encoder part, we replace ordinary convolution with mixed strip convolution; at the bottleneck of the encoder and decoder, the GMAM is combined to convert the encoder and decoder features. This improves the accuracy of ice segmentation and enables precise recognition of ice thickness. Our main contributions are as follows:

(1) An ice-covered transmission lines dataset is constructed for image segmentation.
(2) A Mixed Strip Convolution Module is proposed to extract features of ice-covered transmission lines at different scales.
(3) A global micro awareness module is proposed to improve the model’s encoder and decoder feature conversion ability.
(4) A network based on global micro strip awareness is designed for ice thickness recognition, which can achieve 96.4% mIoU and a 98.1% F1-Score on the ice-covered dataset, and the ice thickness recognition error is controlled within 3.8%.

## 2. Global Micro Strip Awareness Net (GMSA-Net)

**Figure 1:**
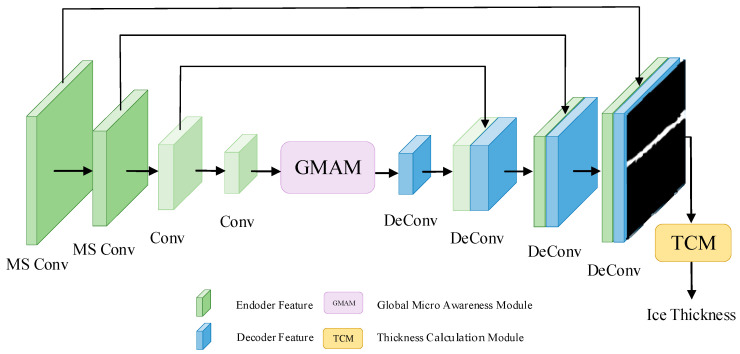
The structure of our proposed GMSA-Net.

The ice thickness identification model for transmission lines designed in this article is shown in Figure 1. In the first step, one obtains the ice-covered image of the transmission line with a camera, and then use an encoder and decoder for feature extraction and segmentation. The encoder extracts features of ice-covered transmission lines through MSCM, and then uses GMAM to perform global and micro interactions of advanced semantic information, converting it. The next step is to generate segmented images through a decoder, and finally recognize the ice thickness through a thickness calculation module.

### 2.1. Image Preprocess

Due to the large pixel size of the images that are directly captured of ice-covered transmission lines, they cannot be used directly for training an image segmentation model or for testing the model. Therefore, it is necessary to preprocess and adjust the image size of the ice-covered images. After we obtain the ice-covered image, we uniformly adjust it to a size of 512 × 512 and perform preprocessing operations such as histogram equalization to make it suitable for training a deep learning model.

### 2.2. Image Segmentation

The division of the ice-covered area of transmission lines is a prerequisite for calculating the ice thickness. Only by ensuring the accuracy of ice-covered area segmentation can accurate ice thickness be obtained. Therefore, we propose an image segmentation model that is based on global micro strip awareness and employs an encoder–decoder architecture for segmenting ice-covered areas. By designing the encoder specifically, the MSCM is proposed to adapt to the shape characteristics of transmission lines. We also designed the GMAM to enhance the semantic information conversion capability at the bottleneck.

#### 2.2.1. Mixed Strip Convolution Module (MSCM)

**Figure 2:**
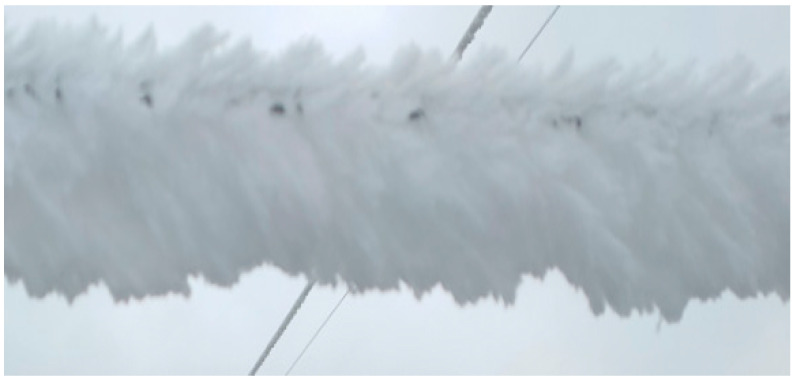
The ice-covered transmission lines.

Ordinary convolution is a square convolution with consistent length and width. When ordinary convolution is used to detect strip objects such as ice-covered transmission lines, it can easily cause the detection area to contain background parts, resulting in redundant extracted image features. In response to this issue, strip convolution is more suitable for detecting such strip objects than ordinary convolution [33]. Strip convolution breaks down ordinary convolutions, such as dividing a 3 × 3 convolution block into 1 × 3 and 3 × 1, which not only improves the convolutional neural network’s ability to extract features of ice-covered transmission lines but also effectively reduces computational parameters. We propose a mixed strip convolution to replace the ordinary convolution in the encoder. Figure 2 is a schematic diagram of the ice-covered transmission lines.

**Figure 3:**
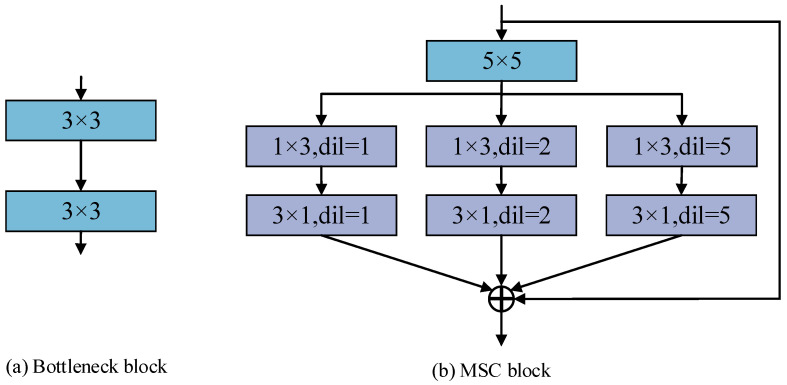
MSC block and bottleneck block.

Due to the irregular ice on the surface of transmission lines, convolutional kernels with different receptive fields are needed to adapt to this situation. We designed strip convolutions with different receptive field sizes, and in order to not significantly increase the number of parameters, we used strip convolutions with consistent kernel sizes and different void rates to expand the receptive field range. Figure 3 shows a schematic diagram of the mixed strip convolution (MSC) block and bottleneck block.

As illustrated in Figure 3, an ordinary convolution of 5 × 5 is used to enhance the convolution channel. Then, 1 × 3 and 3 × 1 convolutions are used, respectively. Additionally, 1 × 3 and 3 × 1 convolutions with a void ratio of 2, and 1 × 3 and 3 × 1 convolutions with a void ratio of 5 are used to extract features of the ice-covered transmission line in parallel. Finally, the image features of different receptive fields are fused together, and residual connections are performed. Due to the fact that the first two convolutional blocks extracted by the encoder are mostly low-level image features such as edges and corners, while the latter convolutional blocks are abstract semantic features, we will replace the first two convolutional blocks in the encoder section with mixed strip convolutional blocks, while the remaining convolutional blocks remain unchanged. When the decoder generates segmented images, the last two up-sampling modules are the key modules for generating the ice-covered area, and cross-layer connections are used to concatenate the mixed strip convolution blocks with the up-sampling modules, guiding these two up-sampling modules to generate segmented images, which helps to improve the segmentation accuracy of the ice-covered area.

#### 2.2.2. Global Micro Awareness Module (GMAM)

**Figure 4:**
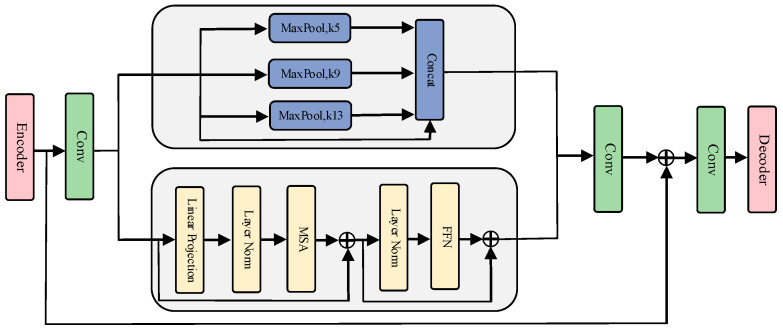
Global micro awareness module.

The bottleneck is located between the encoder and decoder, responsible for converting the semantic information generated by the encoder, removing irrelevant information, and retaining useful semantic features for the decoder [34]. Therefore, this region plays a crucial role and is related to the accurate generation of subsequent segmented images. The features obtained from the encoder require further exploration of their internal connections. Therefore, we propose the GMAM for global and local interaction of features. The GMAM consists of a micro awareness part and a global awareness part. The micro awareness part uses the spatial pyramid pooling (SPP) module to interact with local information, while the global awareness part uses self-attention to interact with semantic features globally. Figure 4 is a schematic diagram of the global micro awareness module.

In the first step, the feature dimension is transformed through convolutional layers. The feature channel dimension at the bottleneck is the largest part of the entire model. So, we reduce the channel dimension to obtain more compact feature information, which is also beneficial for accelerating model training. Then, micro and global awareness are performed separately. Feature pyramid pooling aggregates semantic information locally through pooling layers of different sizes, then performs cascading fusion, and finally recovers to the original dimension through convolutional layers. Through the micro awareness section, we further extract local detail information of encoder features. Our global awareness module uses a self-attention mechanism for awareness, which can learn the global relationships between features. The core component is the multi head self-attention module. The global awareness part divides the features into uniformly sized image blocks, adds learnable position vectors, and adds the image vectors to the position vectors. The image blocks divided in this way have positional information. The specific calculation formula is as follows:

(1) $$Z_{0}=\left[X_{p}^{1};\;X_{p}^{2};\;\ldots ;\;X_{p}^{N}\right]+E_{pos}$$

$X_{p}^{1}$ is the image block, and $E_{pos}$ represents the position vector. Then, global self-attention is calculated, and the attention representation between variables is computed through the utilization of three learning variables: *Q*, *K*, and *V*. The calculation formula is as follows:

(2) $$Head_{j}=Attention\left(Q,\;K,\;V\right)=softmax\left(\;QK^{T}∕\sqrt{d}\;\right)V$$

(3) $$MHA\left(Q,\;K,\;V\;\right)=Concat\left(Head_{1},\ldots ,Head_{Z}\right)w^{o}$$

$w^{o}$ is the weight of multi head self-attention, which is restored to its original size after self-attention calculation is completed. After performing micro awareness and global awareness, we first upscale our respective channel dimensions and restore them to their original feature dimensions. Then, the original input is added to the micro awareness features and global awareness features, and information aggregation is achieved through convolutional layers to obtain the output of the GMAM.

### 2.3. Calculation of Ice Thickness on Transmission Line

The segmentation image of an ice-covered transmission line is essentially a binary image, with the foreground representing the ice-covered area with a pixel value of 255, while the background has a pixel value of 0. Therefore, the pixel values of the ice-covered area can be calculated to obtain the area of the ice-covered area in the image. Based on the pre-measured cable thickness and other parameters, the ice thickness can be obtained by calculating the pixel area difference before and after ice coverage. The calculation formula is

(4) $$h=\frac{1}{2}\left(\frac{D_{1}d_{2}}{d_{1}}-D_{1}\right)$$

$D_{1}$ is the actual diameter of the non-ice-covered transmission line; $d_{1}$ is the number of pixels in the transmission line image of the non-ice-covered line; $d_{2}$ is the number of pixels in the transmission line image of the ice-covered line; h is the ice thickness.

## 3. Experimental Results and Analysis

The dataset used in this experiment was collected internally, and real power transmission cables were built in actual environments. After natural snowfall events, cameras were used to take photos. After data cleaning, a total of 818 images of ice-covered transmission lines were obtained, which were manually annotated and organized into an experimental dataset. The dataset was divided into training and testing sets in an 8:2 ratio. The deep learning framework used in the experiment was Python. The GPU was RTX4090, and the operating system was Ubuntu 20.04.6. The input image size was set to 512 × 512, with a batch size of 16, and 1600 iterations. The Stochastic Gradient Descent (SGD) optimizer was used to update the model parameters.

The segmentation evaluation indicators used in this article are mIoU and F1-Score. The intersection and union ratio refers to the ratio of the intersection and union of the true value and the predicted value. The average intersection and union ratio is the average value of all categories of *IoU*. The specific formula for *IoU* is as follows:

(5) $$IoU=\frac{TP}{FN+TP+FP}$$

TP represents the true value as true and the predicted value as true, FN represents the true value as true and the predicted value as false, and FP represents the true value as false and the predicted value as true. The calculation formula for *mIoU* is

(6) $$mIoU=\frac{1}{N}\Sigma \frac{TP}{FN+TP+FP}$$

N is the predicted category. *F*1-Score is an evaluation parameter that balances precision and recall. The specific formula is as follows:

(7) $$F1-Score=2\times \frac{Precision\times Recall}{Precision+Recall}$$

(8) $$Precision=\frac{TP}{TP+FP}$$

(9) $$Recall=\frac{TP}{TP+FN}$$

### 3.1. Ablation Experiment

We conducted ablation experiments to verify the effectiveness of each part of the proposed GMSA-Net in this article. A total of three comparative experiments were set up. Experiment one was the traditional U-Net model, and Experiment two was to remove the global micro awareness module to verify the effectiveness of the MSCM. Experiment three removed the mixed strip convolution and verified the global micro awareness module. The experimental results are shown in Table 1.

According to Table 1, the traditional U-Net model was not designed for ice-covered transmission lines and achieved the lowest results. Experiment two only added mixed strip convolution, and the mIoU and F1-Score improved by 0.3% and 0.1%, proving that this module can enhance the feature extraction ability for ice-covered transmission lines. Experiment three validated the global micro awareness module, and the experimental results improved compared to traditional U-Net, indicating that the GMAM performed more complex transformation of semantic information at the bottleneck, thereby improving segmentation accuracy. Finally, the model proposed in this article combines the above modules, achieving an mIoU of 96.4% and an F1-Score of 98.1%. In addition, we analyzed the precision and recall of ice-covered area segmentation. From the table, it can be seen that the precision of each experiment did not show significantly differences, while the recall rate of the GMSA-Net undoubtedly achieved a significant lead. A higher precision and recall rate indicate that our model can accurately identify ice-covered areas and has a lower rate of missed detections.

### 3.2. Experiment on the Number of Mixed Strip Convolution Blocks

**Figure 5:**
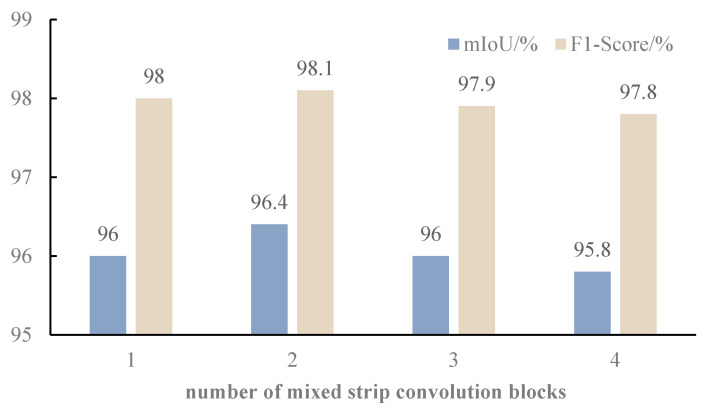
Experimental results of mixed strip convolution blocks.

This article proposes a mixed strip convolution to enhance the feature extraction ability of the encoder. Strip convolution is suitable for extracting low-level features. In order to find suitable replacement positions, mixed strip convolution experiments were conducted. The experimental results are shown in Figure 5.

Figure 5 shows the experimental results. When the number of encoder strips is two, the segmentation effect is optimal. When replacing all the encoders with mixed strip convolution, the experimental effect is not good. The features extracted by deeper convolutional blocks become abstract high-level semantic features, which are not suitable for strip convolution. Therefore, this article sets the first two convolutional blocks of the encoder as mixed strip convolutions.

### 3.3. Feature Pyramid Pooling Experiment

**Figure 6:**
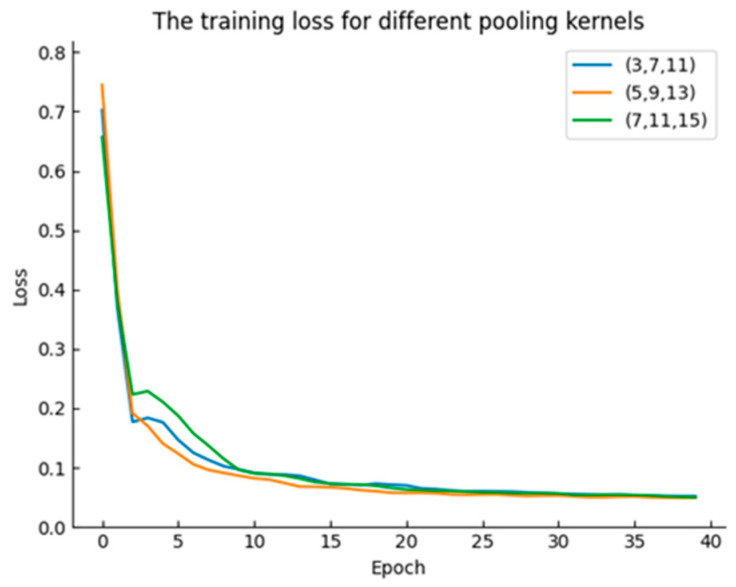
Training loss with different pooled kernels.

The micro part of the GMAM uses feature pyramid pooling for awareness, and obtains multi-scale local information aggregation by setting different pooling kernel sizes. Therefore, experiments were conducted on pooling kernels of different sizes to select appropriate combinations for micro awareness. The training loss for different pooling kernels is shown in Figure 6.

Figure 6 shows the experimental results, where the training loss curve with combinations of (5,9,13) decreases the fastest and the segmentation effect is the best. This combination takes into account large, medium, and small scales, thus obtaining the most comprehensive local feature information. Subsequent experiments will set the feature pyramid pooling to (5,9,13).

### 3.4. Comparison Experiments with Other Algorithms

This section compares the detection performance of other algorithms on ice-covered datasets with the model proposed in this paper. The experimental results are shown in Table 2.

Among them, U-Net, DeeplabV3 [35], and U-Net++ [36] are all convolutional neural network architectures, while TransBTS [37] is a convolutional neural network with a visual attention model. From the table, it can be seen that compared with the CNN model or the CNN+VIT model, GMSA-Net achieved the highest segmentation performance through the MSCM and the GMAM. The high precision and recall rate indicate that our model can accurately identify ice-covered areas, proving the effectiveness of the model.

**Figure 7:**
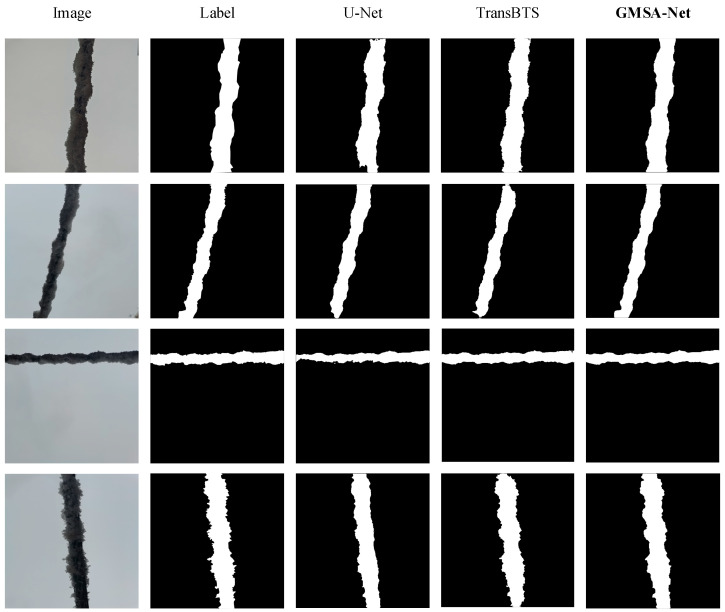
Segmentation effect of different models.

Figure 7 shows a schematic diagram of the segmentation effect of different models. From the figure, it can be seen that the U-Net model exhibits inaccurate edge recognition during segmentation, resulting in some areas of ice cover not being recognized. Although TransBTS performs better than U-Net, there are also some false detection areas. The segmentation effect of our model is closest to the real label, and it can fully recognize the ice-covered area.

### 3.5. Method Validation in Complex Backgrounds

**Figure 8:**
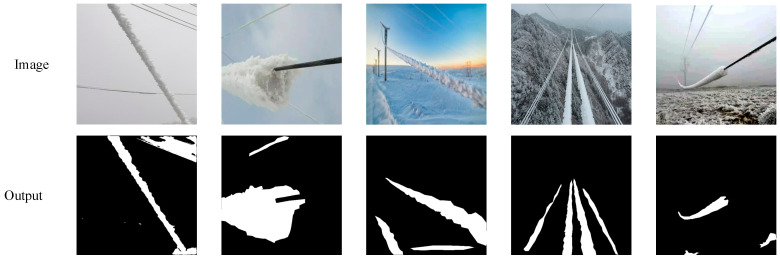
Test results in complex backgrounds.

Because transmission lines is in an overhead state, the background is often very complex in the real world, and it frequently has a large number of clouds. Since the transmission lines set up in uninhabited mountains are more vulnerable to the impact of bad weather, the background will also contain some mountains, trees, etc. Therefore, we chose some images of ice-covered power lines with complex backgrounds to test GMSA-Net to verify whether it can accurately divide the ice-covered area. Figure 8 shows the test results.

In Figure 8, we, respectively, tested the model’s ability to segment ice-covered areas under different backgrounds including white snow, dark clouds and hills. It can be seen from the figure that complicated backgrounds had interfered with the ice-covered division of power lines. In the first figure, the model incorrectly identified the power lines in the upper right corner. In the third and fifth images, the model mistakenly identified snow as the ice-covered transmission line. Despite these minor errors, our model was able to identify all ice-covered areas on transmission lines. The test results show that GMSA-Net maintains a high recognition accuracy even in complex backgrounds.

### 3.6. Ice Thickness Experiment on Transmission Line

This article employs image segmentation to separate the ice-covered area from the background area and calculates the ice thickness by calculating the area of the conductor covered by ice compared to the area without ice cover. In addition, we also use the edge detection method for comparison. The edge detection algorithm first identifies the upper and lower edges of the ice-covered transmission line. It then calculates the pixel difference between the upper and lower edges of the ice-covered transmission line, and uses the pixel difference between the upper and lower edges of the non-ice-covered cable to calculate the ice thickness of the transmission line. The Uncertainty-aware Edge Detector (UAED) [38] is one of the best edge detection algorithms at present. It uses EfficientNet to extract the encoded features, then uses U-Net++ to decode them, and uses uncertainty to study the subjectivity and fuzziness of different annotations in edge detection datasets.

The experimental results are shown in Table 3, and the error between the predicted thickness and the actual thickness is below 5%. The maximum error of ice thickness predicted by the edge detection algorithm is 8.4%, which proves that this method can accurately identify the ice thickness of transmission lines.

## 4. Conclusions

We propose an ice thickness recognition method for transmission lines based on GMSA-Net to address the issue of low detection accuracy in existing methods. The segmentation accuracy of the model has been effectively improved by combining the MSCM and GMAM. The experimental results show that this method can accurately segment the ice-covered area and obtain accurate ice thickness recognition results. The mIoU reaches 96.4%, and the F1-Score is 98.1%. The thickness recognition error is within 3.8%. This provides technical support for subsequent deicing work. The next step is to further improve the algorithm, enhance recognition efficiency, and apply it to the deicing of transmission lines to ensure the safe and reliable operation of transmission lines.

## Figures and Tables

**Table 1:** Ablation experiment results.

Method	mIoU/%	F1-Score/%	Precision/%	Recall/%
Backbone	95.7	97.8	96.6	95.6
Mixed Strip Convolution	96.0	97.9	96.7	96.5
Global Micro Awareness Module	96.1	97.9	97.1	96.0
**Global Micro Strip Awareness**	**96.4**	**98.1**	**97.3**	**96.8**

**Table 2:** Comparison of experimental results with other algorithms.

Method	mIoU/%	F1-Score/%	Precision/%	Recall/%
U-Net	95.7	97.8	96.6	95.6
DeeplabV3	95.4	97.6	96.8	95.1
U-Net++	95.9	97.9	96.9	96.0
TransBTS	96.0	97.9	97.1	95.8
**GMSA-Net**	**96.4**	**98.1**	**97.3**	**96.8**

**Table 3:** Ice cover thickness test for transmission lines.

Actual Thickness/mm	Test Method	Predicted Thickness/mm	Error/%
8.50	GMSA-Net	8.44	0.7
	UAED	8.31	2.2
8.24	GMSA-Net	7.92	3.8
	UAED	7.54	8.4
10.42	GMSA-Net	10.28	1.3
	UAED	9.61	7.7
16.58	GMSA-Net	16.14	2.6
	UAED	15.37	7.2

## Data Availability

Data are contained within the article.
